# High Prevalence of *Schistosoma mansoni* in Six Health Areas of – Kasansa Health Zone, Democratic Republic of the Congo: Short Report

**DOI:** 10.1371/journal.pntd.0003387

**Published:** 2014-12-18

**Authors:** Sylvie Linsuke, Sabin Nundu, Sylvain Mupoyi, Rodin Mukele, Faustin Mukunda, Madeleine Mbuyi Kabongo, Raquel Inocêncio da Luz, Jean-Pierre Van Geertruyden, Marc Van Sprundel, Marleen Boelaert, Katja Polman, Pascal Lutumba

**Affiliations:** 1 Department of Tropical Medicine, Université de Kinshasa, Kinshasa, Democratic Republic of the Congo; 2 Department of Epidemiology, Institut National de Recherche Biomédicale, Kinshasa, Democratic Republic of the Congo; 3 Programme National de Lutte contre la Bilharziose et Parasitoses Intestinales, Kinshasa, Democratic Republic of the Congo; 4 School of Public Health, Georgia State University, Georgia, Atlanta, United States of America; 5 Epidemiology and Scocial Medicine – International Health Unit, University of Antwerp, Antwerp, Belgium; 6 Department of Public Health, Unit of Epidemiology and Control of Tropical Diseases, Institute of Tropical Medicine Antwerp, Antwerp, Belgium; 7 Department of Biomedical Sciences, Unit of Medical Helminthology, Institute of Tropical Medicine Antwerp, Antwerp, Belgium; Federal University of Agriculture, Nigeria

## Abstract

School-aged children suffer the most from schistosomiasis infection in sub Saharan Africa due to poverty and limited sanitary conditions. Mapping of disease burden is recommended and there is a need of updating prevalence data which is as old as 20 years in the Democratic Republic of Congo. An epidemiological and parasitological study was carried out in 2011 in the health zone of Kasansa. Six health areas (HA) were included in the study. In each health area, one primary school was selected. School-aged children were screened for *S. mansoni* infection using parallel Kato-Katz and direct microscopy techniques. A total of 335 school-aged children were screened. The average prevalence was 82.7% and ranged between 59.5–94.9%. Four of the six HAs had a prevalence level over 91%. Of all infected children, about half 112 (43.2%) had light parasite density. These results demonstrate that *Schistosoma mansoni* infection is a bigger problem than anticipated and there is an urgent need to implement effective control measures.

## Author Summary

Schistosomiasis (SCH) is a parasitic infection and one gets infected when bathing or in contact with contaminated fresh water bodies like rivers. It has been shown that people living in poor sanitary conditions and especially school-aged children suffer the most from this infection. Mapping is needed in order to control or prevent the disease. However, in the Democratic Republic of the Congo (DRC), surveillance of schistosomiasis is lacking. Previous data on distribution of schistosomiasis may have lost relevance since the disease was last mapped 20 years ago. This study was carried out in 2011 in the health zone of Kasansa, DRC. Six primary schools were visited and stool samples were collected from children attending the 3rd class. The stool samples were analyzed for *S. mansoni* infection. Up to 335 children were recruited and 82.7% were infected. Moreover, in 4 health areas over 91% of the children were infected. Of all infected children, almost half, 112 (43.2%) had light parasite density. These results demonstrate that *Schistosoma mansoni* infection is a bigger problem than anticipated and there is an urgent need to put effective control measures.

## Introduction

Schistosomiasis (SCH) remains a serious public health problem in developing countries with a tropical climate [1]. Globally, 600 million people are estimated to be at risk for schistosomiasis, 200 million are infected of which 85% live in sub-Saharan Africa. Severe morbidity leads to 41 000 deaths each year [2], [3].

School-aged children are an important high-risk group and suffer the most from SCH [4]. This infection can cause severe consequences like nutritional troubles, anaemia, deterioration of growth, impaired cognitive abilities, and irreversible consequences at adult age such as cirrhosis and an increased susceptibility to HIV [5], [6], [7].

The World Health Organization (WHO) has developed recommendations adapted to each country for the mapping and control of this disease. This control strategy focuses on treatment with praziquantel of populations at high risk for morbidity from schistosomiasis. Treatment intervals and target groups vary with the level of endemicity. When school based prevalence levels of schistosomiasis reach 50% or more by parasitological methods, or 30% based on questionnaires on visible haematuria in high-risk areas, all school-aged children should be treated once a year. In moderate areas, school-aged children should be treated once every two years when the prevalence is between 10% and 50% based on parasitological methods or between 1% and 30% based on questionnaires on visible haematuria. Finally, children should be treated twice during their primary-school years in low risk areas when the prevalence is between 1% and 10% based on parasitological techniques [4]. For effective control strategies, it is therefore crucial to know the epidemiological situation in terms of prevalence and intensity of schistosomiasis.

In the Democratic Republic of the Congo (DRC), schistosomiasis has been known to be endemic in certain provinces [8], [9], [10]. The previous surveys showed variable prevalence of schistosomiasis rates that ranged from less than 3.6% up to more than 96.7% [11], [12], [13], [14], [15], however, the current data are more than 20 years ago [11], [16], [13], [17]. Moreover, Rimoin and Hotez have recently stated that there is a particular dearth of schistosomiasis surveillance activity in the DRC. Currently, there are only estimates of SCH disease burdens that are inaccurate due to lack of studies and these authors stressed that there is an urgent need to examine the prevalence of neglected tropical diseases [18]. Therefore there was urgent need to update the data in order to implement effective control strategies against schistosomiasis.

## Materials and Methods

**Figure 1:**
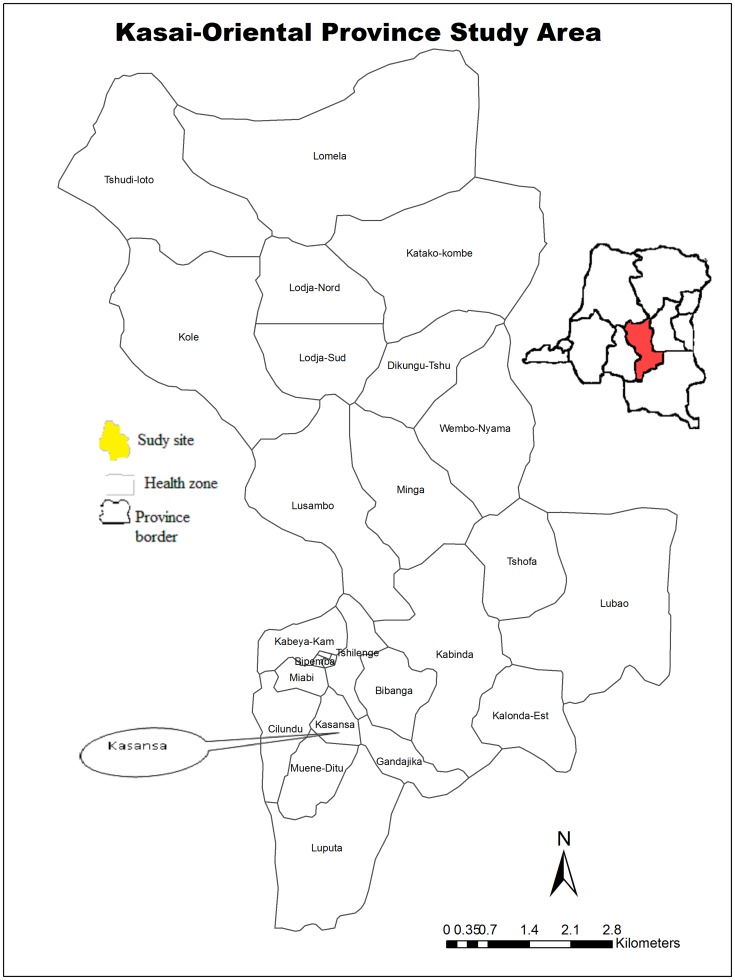
Democratic Republic of the Congo, the province of Kasaï Oriental is highlighted.

**Figure 2:**
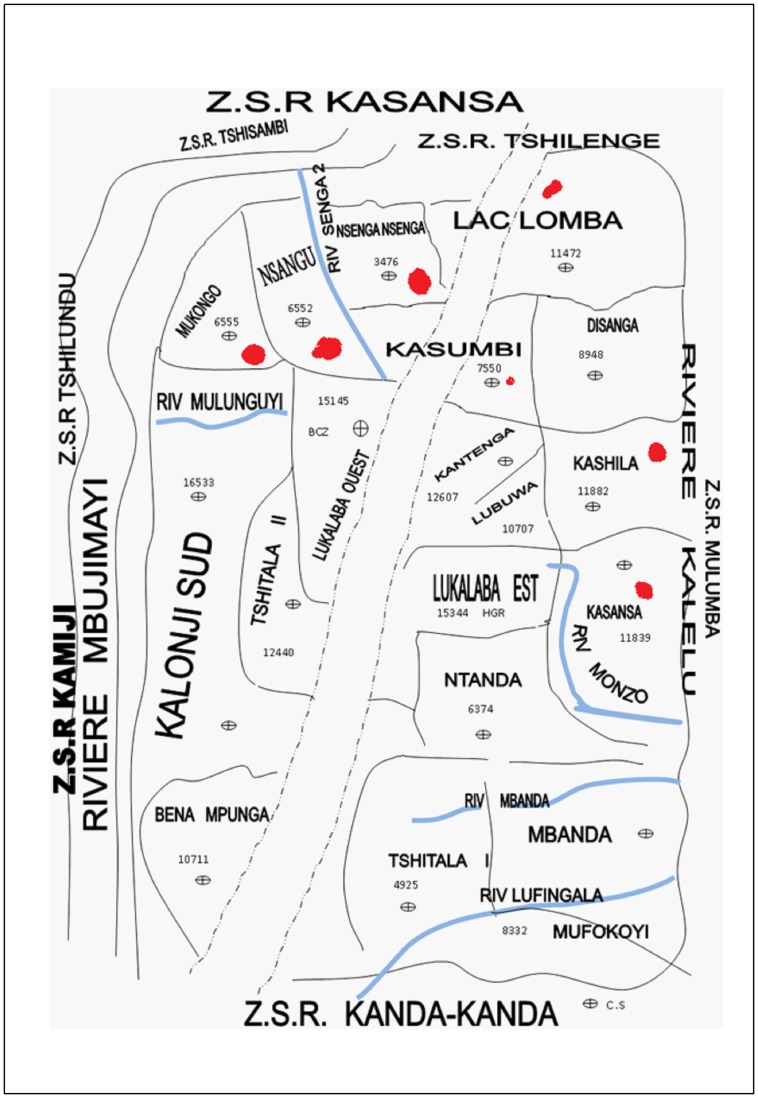
The health zone of Kasansa, red dots indicate the studied health areas.

The pilot survey was carried out between 22nd May and 10th June 2011 in the health zone of Kasansa situated in the province of Kasaï Oriental (Fig. 1), with a humid tropical climate and rainy season lasting from September to April. The HZ of Kasansa has a surface of 2400 Km^2^. The population of this province is estimated at 4.8 million people. Their economic activities are agriculture and hand-crafted exploitation of diamond and the level of poverty reach 62.3% in this area. Several rivers cross this area including: Mulunguyi, Monzo, Nsenga-nsenga, Mbanda Muya, kalelu, Mbuji-Mayi and Lufingala (Fig. 2). In all these rivers, favorable conditions can be found for the reproduction of the intermediate host snails and transmission is favored by the population itself due to their hygienic habits. In the absence of adequate sanitation such as toilettes, latrines and running water, the main water-related activities are done on the shores of these rivers.

The province of Kasaï Oriental is divided into 51 administrative health zones (HZ) and each HZ is further divided into health areas (HA). The main study site was the HZ of Kasansa with estimated 191 986 inhabitants and it is further subdivided into 19 HA. According to the HZ health reports (local health zone reports, unpublished data); cases of SCH were regularly reported in six HA. These were the HA in which the study was conducted: Kasansa, Kashila, Lac-lomba, Mukongo, Nsangu and Nsenga-nsenga as shown in Fig. 2. One school with a minimum of fifty students in the 3rd elementary school class was selected in each HA.

Following WHO guidelines [19] a minimal sample of 200–250 children (8 randomly selected classes) should be selected in the health zone. And for this study, a total of 355 school children were randomly selected in 6 primary schools in each HA and a parasitological baseline survey was performed for the detection of *S. mansoni* infection and intensity. From each child, one stool sample was collected and transferred to the laboratory for examination by Kato-Katz (2×25 mg) and direct microscopy techniques [20], [21]. Two slides were prepared to perform the Kato-Katz while the third slide was prepared for direct microscopy technique. Two slides for kato-katz were examined by two independent readers within 24 hours. The visualized eggs were counted in those two slides and the sum of the egg-count was multiplied by the factor 20 in order to get the number of eggs per gram of feces (EPG). Parasite egg counts were utilized to classify *S. mansoni* infection into light (1-99 EPG), moderate (100–399 EPG) or heavy infections (≥400 EPG) according to WHO guidelines [19].

Data was double-entered and validated in EPI INFO version 3.5.1 software and analysed using STATA version 11.0 (STATA Corp, Lakeway, College Station, Texas, USA).

### Ethics statement

Ethical approval was provided by the Ethical committees of the University Hospital, Antwerp, Belgium (reference number: 10/36/237) and the School of Public Health, Kinshasa University, DRC (approbation number: ESP/CE/079/10).

Before inclusion, written informed consent was obtained from the parents or legal guardians. Each informed consent was signed. For those who did not know how to sign for any reason, we took a thumb print instead of the signature. At the end of the study, children who were tested positive for *S. mansoni* were treated according to the WHO guidelines [4] with 40 mg/kg body weight of praziquantel (Distocide 600 mg) manufactured by Shin Poong Pharmaceuticals, Seoul Republic of Korea.

## Results

In total, three hundred and thirty five (335) children were included in the study. Median age was 11 years old (IQR  = 2 with a minimum of 8 and maximum of 16 years old) and 56.4% of the study population (n = 189) were males.

The average prevalence of *schistosomiasis* was extremely high (82.7%; 95% CI: 78.6–86.7%) in the health study area. Looking at the prevalence by HA, 94.9% (95% CI: 82.7–99.4%), 94.9% (95% CI: 82.7–99.4%), 92.0% (95% CI: 80.8–97.8%), 91.0% (95% CI: 83.1–96.0%), 74.5% (95% CI: 59.7–86.1%), 59.5% (95% CI: 47.4–70.7%) of children were positive to infection in the HA of Nsangu, Mukongo, Nsenga-Nsenga, Kasansa, Kashila and Lac-Lomba respectively (Table 1). Among these infected children, 112 (43.2%) were found to have a light infection while 83 (32.0%) had a moderate infection and only 64 (24.7%) children presented a heavy infection. Of the 335 participants, 88.1% reported the presence of a latrine in the household. The presence of latrines in the household did not influence the prevalence of SCH in these children; the prevalence was similar to those that did not own a latrine (Table 1).

**Table 1:** Prevalence of *S. mansoni* in the six health areas of Kasansa health zone.

Variables	Prevalence of *S. mansoni*		
	n	n of positive	% (IC95%)
Number total of participants (335)		277	82.7 (78.6–86.7)
**Sex**			
M	189	165	87.3 (81.7–91.7)
F	146	112	76.7 (69.0–83.3)
**Presence of latrine in the household**			
No	40	34	85.0 (73.4–96.5)
Yes	295	243	82.4 (78.0–86.7)
**Health areas**			
Kasansa	89	81	91.0 (83.1–96.0)
Kashila	47	35	74.5 (59.7–86.1)
Lac-Lomba	74	44	59.5 (47.4–70.7)
Mukongo	36	34	94.4 (81.3–99.3)
Nsangu	39	37	94.9 (82.7–99.4)
Nsenga-Nsenga	50	46	92.0 (80.8–97.8)

## Discussion

This descriptive study showed that schistosomiasis is a real public health problem in six HA of Kasansa HZ. The extreme high SCH average prevalence observed in this study area is similar to the prevalence reported in other provinces of the country [22], [23]. This situation could be explained by the geographical setting of Kasansa HZ as shown in Fig. 2. Indeed, there are seven rivers and intense contact of the water of these rivers for domestic needs. Moreover, no control measures have been taken in this HZ that could have reduced the prevalence [2].

Furthermore, it was observed that quite a large proportion of the children (43.2%) were carrying a heavy infection. This situation might be explained by the dynamics of schistosomiasis transmission which is favored in the study area of this health zone by the geographical situation of this area [24]. In 1950 Rodhein et al., cited by Janssens et al. reported that the territory of Tshilenge in the province of Kasaï was an endemic zone for schistosomiasis and found that the population had an infestation indice varying from 6 to over 60% [25]. Consequently it is noticed that the situation remained constant over 50 years later. This indicates that there is an urgent need to establish adequate control measures to sustainably diminish the disease burden.

When the distribution of SCH is evaluated by HA, the study shows some geographical variability which can be explained by the focal character of schistosomiasis. While the prevalence in all six HA is more than 50%, the HA of Lac-Lomba presented the lowest prevalence (59.5%; 95% CI: 47.4–70.7%) compared to the other HA (Table 1). Unfortunately, this study did not evaluate the factors associated to infection that could explain this difference. However, the fact that the HA such as Kasansa, Mukongo, Nsangu and Nsenga-Nsenga have rivers passing through these areas might explain that the children living here are more closely in contact with these rivers and therefore more at risk and/or infected compared to children living in Lac-Lomba. Risk factors such as proximity to water bodies [24], [26], and body contact with contaminated water were not taken into account in this pilot survey. Owning a latrine did not significantly reduce the prevalence of *S. mansoni* infection (Table 1). It leads to the suggestion that the latrines, although widely present, are not properly used and that the children are most likely infected while bathing in the rivers.

In conclusion, the school-aged children living in Kasansa, Mukongo, Nsangu, Nsenga-Nsenga and Lac-Lomba health areas of Kasansa HZ highly infected by intestinal schistosomiasis, this situation demands urgent implication of national and local authorities to implement schistosomiasis control activities.

## Supporting Information

S1 Dataset **The dataset of this study.**
(XLS)

S1 Checklist **The Strobe checklist linked to this study.**
(PDF)
