# Supplementary material for: High Prevalence of Schistosoma mansoni in Six Health Areas of – Kasansa Health Zone, Democratic Republic of the Congo: Short Report
Source: PLoS Negl Trop Dis. 2014 Dec 18;8(12):e3387. doi: 10.1371/journal.pntd.0003387 (PMC4270747; doi:10.1371/journal.pntd.0003387)
Supplement: S1 Checklist — The Strobe checklist linked to this study. (PDF) [file pntd.0003387.s002.pdf]

STROBE Statement—Checklist of items that should be included in reports of ***cross-sectional studies***

	Item No	Recommendation
**Title and abstract**	1	(*a*) Indicate the study's design with a commonly used term in the title or the abstract<br>in the abstract: Epidemiological and parasitological study was carried out in 2011
		(*b*) Provide in the abstract an informative and balanced summary of what was done and what was found<br>Epidemiological and parasitological study was carried out in 2011 in Health zone of Kasansa in Democratic Republic of Congo. Six health areas were included in the study. In each health area, one primary school was selected. Kato-Katz and direct microscopy examinations were performed in school-aged children.<br>High Schistosoma prevalence levels (82.7%) were found in the Health Zone of Kasansa and certain study areas presented prevalence levels reaching nearly 100%. Of all infected children, the majority or 127 (45.9%) had light parasite density.
**Introduction**		
Background/rationale	2	Explain the scientific background and rationale for the investigation being reported<br>School-aged children are an important high-risk group and suffer the most from Schistosomiasis. For effective control strategies, there is a need of knowledge of epidemiology situation in terms of prevalence and intensity of schistosomiasis. The current data of Democratic Republic of the Congo (DRC) are more than 20 years old. Moreover, Rimoin and Hotez have recently stated that there is a particular dearth of Schistosomiasis surveillance activity in the DRC. Currently, there are only estimates of SCH disease burdens that are inaccurate due to a lack of studies and these authors stressed that there is an urgent need to examine the prevalence of neglected tropical diseases
Objectives	3	State specific objectives, including any prespecified hypotheses<br>To update epidemiologic data in order to formulate new effective control strategies against schistosomiasis.
**Methods**		
Study design	4	Present key elements of study design early in the paper<br>A pilot school-based study was carried out
Setting	5	Describe the setting, locations, and relevant dates, including periods of recruitment, exposure, follow-up, and data collection<br>The survey was carried out between 22nd May and 10th June 2011 in the Health Zone of Kasansa situated in the province of Kasaï Oriental. The province of Kasaï Oriental counts 51 administrative Health Zones (HZ) and each HZ is further divided into Health Areas (HA). The main study site was the HZ of Kasansa with estimated 191 986 inhabitants. According to the HZ health reports, cases of SCH were regularly reported in six HA. These were the HA chosen to conduct the study: Kasansa, Kashila, Lac-lomba, Mukongo, Nsangu and Nsenga-nsenga. a parasitological baseline survey was performed for the detection of *S. mansoni* infection and intensity. From each child, one stool sample was collected and transferred to the laboratory for examination by Kato-Katz (2X25 mg) and direct microscopic examination.
Participants	6	(*a*) Give the eligibility criteria, and the sources and methods of selection of participants<br>The main study site was the HZ of Kasansa with estimated 191 986 inhabitants. According to the HZ health reports, cases of SCH were regularly reported in six HA. These were the HA chosen to conduct the study: Kasansa, Kashila, Lac-lomba, Mukongo, Nsangu and Nsenga-nsenga. One school with minimum fifty students in

		the 3rd elementary school class was selected in each HA.<br>For this study, a total of 355 school children were randomly selected in 6 primary schools in each HA.
Variables	7	Clearly define all outcomes, exposures, predictors, potential confounders, and effect modifiers. Give diagnostic criteria, if applicable<br>Two slides were prepared to perform the Kato-Katz while the third slide was prepared for direct wet mount examination. The visualized eggs were counted in each slide (Kato-Katz and wet mount) and the sum of the eggcount was multiplied by the factor 24 order to get the number of eggs per gram of feces (EPG). Egg-counts were utilized to classify infections into light (1-99 EPG), moderate (100- 399 EPG) or heavy infections (≥ 400 EPG) according to WHO standards.
Data sources/ measurement	8*	For each variable of interest, give sources of data and details of methods of assessment (measurement). Describe comparability of assessment methods if there is more than one group<br>The slide readings are the source of the data; see answer nr 7
Bias	9	Describe any efforts to address potential sources of bias<br>Not applicable
Study size	10	Explain how the study size was arrived at<br>Following WHO guidelines (12) a minimal sample of 200-250 children (8 randomly selected classes) should be selected in the Health Zone. And for this study, a total of 355 school children were randomly selected
Quantitative variables	11	Explain how quantitative variables were handled in the analyses. If applicable, describe which groupings were chosen and why<br>Egg-counts were utilized to classify infections into light (1-99 EPG), moderate (100- 399 EPG) or heavy infections (≥ 400 EPG) according to WHO sandards.
Statistical methods	12	(*a*) Describe all statistical methods, including those used to control for confounding<br>Not applicable.
		(*b*) Describe any methods used to examine subgroups and interactions<br>Not applicable
		(*c*) Explain how missing data were addressed<br>Children that did not present a stool sample were excluded from the study
		(*d*) If applicable, describe analytical methods taking account of sampling strategy<br>Random selection of children in the 3rd elementary school class in the six pre-selected Health Areas
		(*e*) Describe any sensitivity analyses<br>Not applicable
**Results**		
Participants	13*	(a) Report numbers of individuals at each stage of study—eg numbers potentially eligible, examined for eligibility, confirmed eligible, included in the study, completing follow-up, and analysed<br>335 children were included in the study
		(b) Give reasons for non-participation at each stage<br>Not providing a stool sample or not obtaining a written informed consent resulted in non-participation
		(c) Consider use of a flow diagram Not applicable
Descriptive data	14*	(a) Give characteristics of study participants (eg demographic, clinical, social) and information on exposures and potential confounders<br>Median age was 11 years old (IQR=2 with a minimum of 8 and maximum of 16 years old) and 56.4% of the study population (n=189) were male.
		(b) Indicate number of participants with missing data for each variable of interest

		No missing values
Outcome data	15*	Report numbers of outcome events or summary measures<br>This was a cross-sectional study where all participants underwent parasitological examination
Main results	16	(*a*) Give unadjusted estimates and, if applicable, confounder-adjusted estimates and their precision (eg, 95% confidence interval). Make clear which confounders were adjusted for and why they were included<br>The overall prevalence of S. mansoni infection was extremely high 82.7% (95% CI: 78.6-86.7%).
		(*b*) Report category boundaries when continuous variables were categorized<br>Not applicable
		(*c*) If relevant, consider translating estimates of relative risk into absolute risk for a meaningful time period<br>Not applicable, no risk analysis applied
Other analyses	17	Report other analyses done—eg analyses of subgroups and interactions, and sensitivity analyses<br>Not applicable
**Discussion**		
Key results	18	Summarise key results with reference to study objectives<br>Extreme high iSCH prevalences, sometimes near 100% in certain Health Areas (see table 1).
Limitations	19	Discuss limitations of the study, taking into account sources of potential bias or imprecision. Discuss both direction and magnitude of any potential bias<br>Risk factors such as proximity to water bodies (15,16), and body contact with contaminated water were not taken into account in this pilot survey.
Interpretation	20	Give a cautious overall interpretation of results considering objectives, limitations, multiplicity of analyses, results from similar studies, and other relevant evidence<br>The school-aged children living in Kasansa are very infected by intestinal schistosomiasis. Owning a latrine did not significantly reduce the prevalence of S. mansoni infection (Table 1). It leads to the suggestion that the latrines, although widely present, are not properly used and that the children are most likely infected while bathing in the rivers.
Generalisability	21	Discuss the generalisability (external validity) of the study results<br>This study was a pilot survey in the Kasansa Health Zone to determine the prevalence of Schistosomiasis. The generalisability is applicable for other settings in the DRC that resemble the Kasansa Health Zone.
**Other information**		
Funding	22	Give the source of funding and the role of the funders for the present study and, if applicable, for the original study on which the present article is based<br>Funding was provided by the VLIR UOS Project, Grant Nr 42/FA02000/13/5806.

*Give information separately for exposed and unexposed groups.

Note: An Explanation and Elaboration article discusses each checklist item and gives methodological background and published examples of transparent reporting. The STROBE checklist is best used in conjunction with this article (freely available on the Web sites of PLoS Medicine at http://www.plosmedicine.org/, Annals of Internal Medicine at http://www.annals.org/, and Epidemiology at http://www.epidem.com/). Information on the STROBE Initiative is available at www.strobe-statement.org.
